# Spontaneous Hepatic Hemorrhage as a Rare Presentation of Amyloid Light Chain (AL) Amyloidosis: A Case Report

**DOI:** 10.7759/cureus.76791

**Published:** 2025-01-02

**Authors:** Manjul Srivastava

**Affiliations:** 1 Internal Medicine, Northwest Medical Center, Tucson, USA

**Keywords:** amyloidosis al, factor x deficency, hepatic artery embolization, light chain amyloidosis, spontaneous hepatic subcapsular hematoma

## Abstract

Spontaneous hepatic hemorrhage due to amyloidosis light chain (AL) is a rare but serious life-threatening condition. In this case, a 64-year-old male patient presented to the emergency room with acute right shoulder pain. He was recently diagnosed with type 2 diabetes. He did not have any recent trauma, procedures, history of hepatitis, alcoholism, or chronic inflammatory or bleeding disorders. The patient’s computed tomography (CT) scan of the abdomen was positive for acute subcapular hematoma. He developed acute nephrotic syndrome within two days of admission. Further workup showed elevated monoclonal lambda light chains and confirmed AL amyloidosis on bone marrow biopsy.

This case is unique because subcapsular hepatic hematoma was the only manifestation of AL amyloidosis. While amyloid deposition does occur in systemic amyloidosis, it is exceptionally rare for patients to present with spontaneous hepatic rupture without any prior diagnosis. Recognizing this atypical presentation of AL amyloidosis is crucial for timely intervention and improved outcomes. Familiarity with the rare etiology of intrahepatic hemorrhage may help to decrease the high mortality rate associated with this rare disease. Treatment for hepatic hemorrhage consists of aggressive blood transfusion and CT-guided hepatic artery embolization. Chemotherapy with cyclophosphamide, bortezomib, and dexamethasone (CyBorD) can halt amyloid deposition.

## Introduction

Amyloidosis is caused by the misfolding of the soluble precursor protein, which leads to amyloid deposition in various organs. It primarily affects men, mostly between the ages of 60 and 70. There are different types of amyloidosis, such as immunoglobulin light chain (AL), systemic amyloid (AA), apolipoprotein A1 (Apo A1), transthyretin (ATTR), and beta (β)-amyloidosis related to dialysis [1-2]. Amyloidosis light chain amyloidosis is a lethal form of amyloidosis. This is due to the production of abnormal plasma cells in the bone marrow, which generate abnormal antibodies called monoclonal immunoglobulin light chains [3-5]. Hepatic deposition of amyloid is common but mostly asymptomatic. If it manifests, clinical features are fatigue, unintentional weight loss, and early satiety. This is a unique case report because spontaneous hepatic rupture was the only manifestation of AL amyloidosis. Subcapsular hematoma due to AL amyloidosis is a very rare and underdiagnosed complication leading to fatality [6-9].

## Case presentation

A 64-year-old male patient presented to the emergency room with acute right shoulder pain. He had a medical history of new-onset type 2 diabetes with hemoglobin A1C (HbA1C) of 6.7 mmol/L. He was in his normal state of health until three days ago when he started to develop right shoulder pain. The patient had normal laboratory workup four months prior to his presentation. He tried to reduce the pain by taking Tylenol but had no relief. The pain became increasingly worse to the point that he was no longer able to work. The patient had been trying to lose weight over the past six months given his new diabetes diagnosis and had lost about 30 pounds. There was no history of recent trauma, procedures, hepatitis, alcoholism, chronic inflammation, bleeding, or clotting disorders. On physical examination, he appeared weak and dizzy. His blood pressure was 95/70 (systolic/diastolic) mmHg, and there was a significant drop from his baseline of 130/80 (systolic/diastolic). The right shoulder had no swelling or erythema and was within normal range of movement. Hepatomegaly with severe abdominal tenderness in the right upper quadrant and epigastric region was noted. Neither ascites nor peripheral edema were noted. Initially, his lab results showed anemia, thrombocytopenia, elevated liver enzymes, hyperbilirubinemia, and hypoalbuminemia. His workup for common causes of liver bleed was negative, including hepatitis, malignancy, human immunodeficiency virus, tuberculosis, syphilis, and clotting factor deficiency. Pertinent lab results are shown in Table 1.

**Table 1:** The patient's blood test results

Parameter	Patient’s lab result	Reference value
Hemoglobin	10.6 g/dl	13.5-17 g/dl
Mean corpuscular volume	97 fl	78-100 fl
Mean corpuscular hemoglobin	30.3 pg	27-34 pg
Mean corpuscular hemoglobin concentration	31 g/dl	31-37 g/dl
Leucocytes count	6.3 Kµ/l	4-11 Kµ/l
Platelet count	111 K/µl	130-450 K/µl
Prothrombin time	13.9 seconds	9.5-12.5 seconds
International normalized ratio	1.2	0.9-1
Aspartate transaminase	433 U/L	12-47 U/L
Alanine transaminase	208 U/L	5-60 U/L
Alkaline phosphatase	368 U/L	40-140 U/L
Albumin	2.1 g/dl	3.4-4.9 g/dl
Prealbumin	5.1 mg/dl	20-40 mg/dl
Total protein	4.7 g/dl	6.0-8.0 g/dl
Bilirubin total	2.8 mg/dl	0.2-1.3 mg/dl
Bilirubin direct	1.3 mg/dl	<=0.3 mg/dl
Bilirubin unconjugated	1.5 mg/dl	<=1.3 mg/dl
Factor X (Stuart factor)	78%	50-150
Alfa fetoprotein	2.8 ng/ml	<=8.7 ng/ml
Carcinoembryonic antigen	3.7 ng/ml	<=5.0 ng/ml
Carbohydrate antigen 19-9	3 U/ml	<=35 U/ml
Serum sodium	139mmol/L	134-147 mmol/L
Serum potassium	3.6 mmol /L	3.6-5.3 mmol/L
Serum chloride	103 mmol/L	95-108 mmol/L
Serum bicarbonate	29 mmol/L	19-31 mmol/L
Serum calcium	8.4 mg/dl	8.8-10.43 mg/dl
Serum phosphorus	2.6 mg/dl	2.4-4.8 mg/dl
Serum glucose	102 mg/dl	70-115 mg/dl
Serum blood urea	14 mg/dl	8-25 mg/dl
Serum creatinine	0.76 mg/dl	0.60-1.50 mg/dl
Glomerular filtration rate	74 ml/min/1.73m^2^	>60ml/min/1.73m^2^
Hepatitis A IgM antibody	Negative	Negative
Hepatitis B surface antigen	Negative	Negative
Hepatitis B surface antibody	13.13 mIU/mL	<=9.9 mIU/mL
Hepatitis B core antibody	Nonreactive	Nonreactive
Hepatitis C antibody	0.08 index	<0.79 index
QuantiFERON (QFT)- tuberculosis (TB)	Negative	Negative

**Figure 1:**
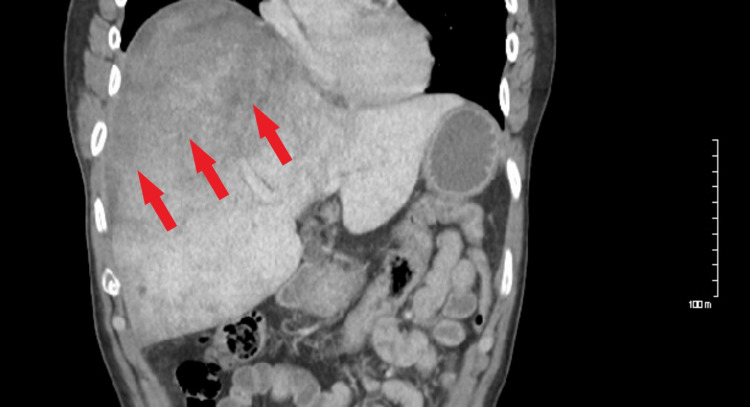
A CT scan shows spontaneous hepatic hemorrhage

A computed tomography (CT) scan was performed, which showed a right subcapsular hematoma of 4.4 cm with extravasation focused at the medial aspect of the hepatic dome (Figure 1). There was also a heterogeneous appearance of the hepatic parenchyma.

The patient was admitted to the surgical intensive care unit (ICU) due to active hepatic bleeding. His blood pressure further dropped to 88/60 mmHg, and the hemoglobin was reduced by three points to 7.6 g/dL. Two units of blood were transfused as he became hemodynamically unstable. He underwent CT-guided hepatic artery embolization; however, he became hemodynamically unstable again, requiring packed red blood cells (RBCs) and fresh frozen plasma (FFP). He underwent another CT-guided hepatic artery embolization, and a 14F drain was placed. He remained anemic with continued elevated liver enzymes. Unfortunately, the hematoma did not resolve. Furthermore, the cause of the liver bleed remained a mystery.

Subsequently, the patient underwent a laparoscopic hand-assisted evacuation of the liver hematoma. His breathing worsened, and a chest X-ray revealed right pleural effusion and collapse of the right middle and right lower lung lobes. A thoracic surgeon recommended video-assisted thoracoscopic surgery (VATS) and decortication to resolve a persistent, loculated large right pleural effusion. The patient further developed an unexplained new-onset nephrotic range proteinuria (Table 2). His labs for anti-DNA, anti-glomerular basement membrane, anti-phospholipase A2 receptor autoantibody (PLA2R), myeloperoxidase, proteinase 3, and antineutrophil cytoplasmic antibodies (ANCA) were all negative. The C3-C4 complement levels were normal.

**Table 2:** The patient's lab results (initial workup for proteinuria)

Parameter	Patient’s lab result	Reference value
Protein urine	300 mg/dl	Negative
Microalbumin normalized to creatinine	5270 mg/g creatinine	<29 mg/g creatinine
Cryoglobulin	Negative	Negative
C3 complement	100 mg/dl	90-180 mg/dl
C4 complement	44 mg/dl	16-47 mg/dl
Anti-nuclear antibody	Negative	Negative
Anticytoplasmic antibody	Negative	Negative
Glomerular basement membrane antibody index	<0.2	<=0.9
Phospholipase A2receptor antibody index	<1:10	<1:10
Myeloperoxidase antibody Index	<0.2	<=0.9
Proteinase-3 antibody index	<1.0	<1.0

As no clearly identifiable cause could be determined for the patient's liver bleeding and with the new onset of unexplained nephrotic range proteinuria, further work-up became crucial. Serum electrophoresis was negative for multiple myeloma and monoclonal gammopathy (Table 3). However, the patient’s serum free light chain (FLC) showed abnormally elevated levels of lambda FLC (233 mg/L) and kappa FLC (30.82 mg/L). The kappa/lambda reduced ratio of 0.13 pointed towards AL amyloidosis.

**Table 3:** The patient's lab results (further workup for unexplained proteinuria) PES: protein electrophoresis

Parameter	Patient’s lab result	Reference value
Alpha 1 PES	0.4 g/dl	0.1-0.4 g/dl
Alpha 2 PES	0.4 g/dl	0.6-1.2 g/dl
Beta PES	0.7 g/dl	0.6-1.3 g/dl
Gamma PES	0.3 g/dl	0.6-1.8 g/dl
Immunoglobulin G	456 mg/dl	694-1618 mg/dl
Immunoglobulin A	276 mg/dl	81-463 mg/dl
Immunoglobulin M	15 mg/dl	48-271 mg/dl
Protein normalized to creatinine	6966 mg/g creatinine	15-170 mg/g creatinine
Kappa free light chain	30.82 mg/l	3.30-19.40 mg/l
Lambda free light chain	233.44 mg/l	5.71-26.30 mg/l
Kappa/lambda free light chain	0.13	0.26-1.65

**Figure 2:**
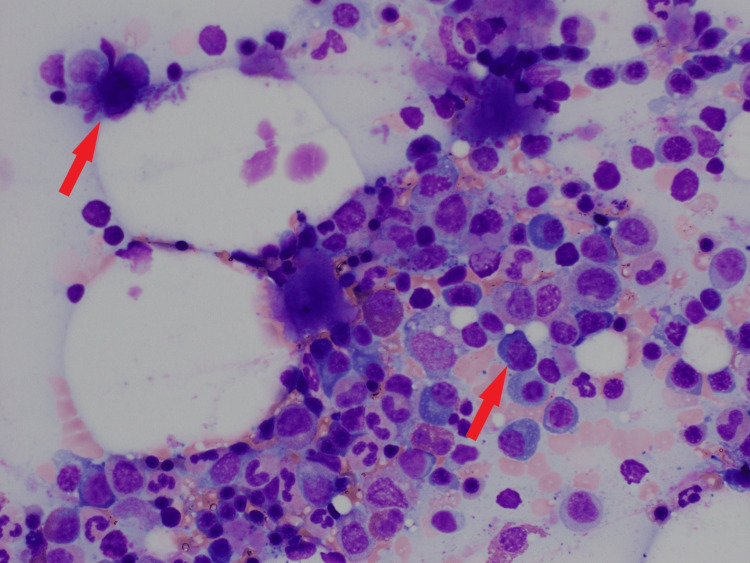
Bone marrow aspirate smear with 8% plasma cells identified (400x magnification)

 The patient underwent a bone marrow biopsy which showed 8% of plasma cells (Figure 2).

**Figure 3:**
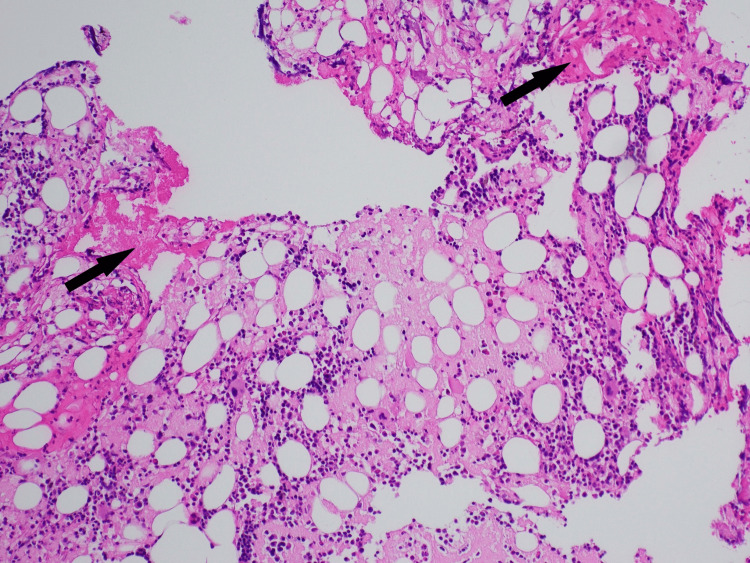
Bone marrow core biopsy with amyloid (100x magnification)

Congo red staining was positive for amyloid deposits (Figure 3).

**Figure 4:**
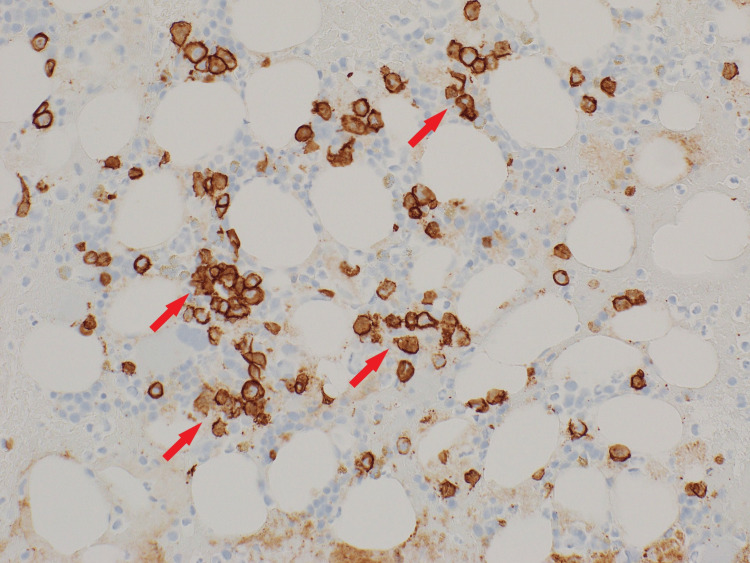
Clot section with CD138 positive immunohistochemical staining in 10%-20% of marrow cells (200x magnification)

CD138 malignant plasma cells were positive in 10%-20% of the marrow cells (Figure 4). 

Fluorescent in situ hybridization (FISH) showed chromosomal abnormalities, deletion 13q14.3, and monosomy 13. There was also translocation of chromosomes (11;14).

A liver biopsy was not done because of bleeding risk due to subcapsular hepatic hematoma. The patient was diagnosed with lambda light chain amyloidosis with renal and hepatic involvement. The cause for elevated lambda FLC was confirmed by Congo stain-positive amyloid and abnormal CD138-positive plasma cells on the bone marrow biopsy. His echocardiogram had no interventricular septal thickening or features of restrictive cardiomyopathy. Cardiac biomarkers troponin and NT-pro BNP were within normal range.

**Figure 5:**
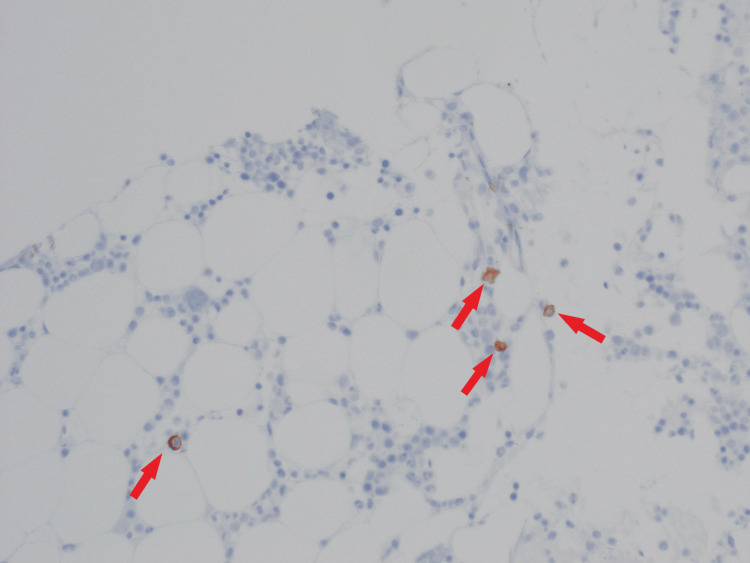
Clot section with CD138 positive immunohistochemical staining in 1%-2% of marrow cells (200x magnification)

Treatment of AL amyloidosis with cyclophosphamide, bortezomib, and dexamethasone (CyBorD) was initiated after a multidisciplinary discussion. This was based on the ANDROMEDA study. The patient was in remission after six cycles. Figure 5 shows the reduction of CD138-positive plasma cells down to 1%-2% from earlier 10%-20%. The patient continues on daratumumab every four weeks as maintenance therapy.

## Discussion

In AL amyloidosis there is a generation of abnormal plasma cells that express the CD138 marker. These cells produce abnormal antibodies called light chains, which misfold and deposit amyloid in various organs like the kidney (46%), heart (30%), liver (9%), spleen (5%-10%), and gastrointestinal tract (7%) [6]. Deposition in the liver tissue is seen mostly in the perisinusoidal space, parenchyma, portal vessels, central vein, portal stroma, and intercellular space [2]. Hepatic amyloidosis is mostly asymptomatic. When it becomes symptomatic, it commonly presents as unintentional weight loss, fatigue, early satiety, hepatomegaly, cholestasis, portal hypertension, and proteinuria. Nonspecific presentation of AL amyloidosis leads to significant diagnostic challenges. Liver functions may or may not be deranged [1-2, 5-6, 10-15]. Amyloidosis light chain amyloidosis has a poor survival time of less than nine months if left untreated [2, 16]. Therefore prompt diagnosis and treatment are critical.

Spontaneous rupture of the liver is a rare, life-threatening presentation of AL amyloidosis [2,4, 8-9,17-18]. Death due to spontaneous hepatic rupture is within 1-2 weeks [19]. As such, time is crucial, and delay in treatment increases fatality. Literature review shows there have been only seven such cases with no known systemic AL amyloidosis who presented with spontaneous hepatic bleeding; among these cases, there have been only three survivors [1]. The patient in this case study is the fourth known survivor. Recognizing this atypical presentation is critical for ensuring better outcomes. If common causes of liver bleeding like trauma, hepatocellular carcinoma, hepatic adenoma, preeclampsia, peliosis hepatitis, systemic lupus erythematosus, and clotting factor deficiency like Factor X are ruled out, evaluation of AL amyloidosis is necessary. Familiarity with this rare etiology of intrahepatic hemorrhage should help to reduce the disease’s high mortality rate. Acquired deficiency of Factor X is the most common coagulation factor deficiency that has been identified in patients with AL amyloidosis [1,19]. The deficiency of this factor, along with increased blood vessel fragility and impaired vasoconstriction, can lead to massive hemorrhage and even death. This is not always true, as in our patient, Factor X was normal. Computed tomography scans, MRIs, and ultrasounds can help diagnose intrahepatic hemorrhage [1-2,7,9,14,17-19]. Stabilizing the patient is important with blood transfusion and fresh frozen plasma. Computed tomography-guided hepatic angiographic embolization is used to stop the bleeding [2,7,9,14]. A liver transplant is recommended only if there is progressive liver failure. Initial diagnosis for AL amyloidosis is done by serum and urine electrophoresis to evaluate for any associated multiple myeloma or monoclonal gammopathy. The serum FLC assay for kappa and lambda immunoglobulin chain levels is the first screening test. Subcutaneous fat aspirate biopsy and bone marrow biopsy help to confirm the diagnosis. Congo red staining of the amyloid fibril is the gold standard for the diagnosis of amyloidosis [1,10-11]. Fluorescent in situ hybridization studies are done to determine associated cytogenetic abnormalities. Amyloidosis light chain amyloidosis is often associated with the translocation of chromosomes (11;14) when genes are exchanged between chromosomes 11 and 14. This leads to abnormal cell proliferation due to overexpression of the cyclin D1 oncogene. Another abnormality commonly associated with AL amyloidosis is the deletion of a specific region of chromosome 13 (13q14.3) or monosomy 13 when there is a complete loss of one entire chromosome. These have a higher risk for disease progression and poor prognosis. Mass spectrometry is another gold standard that is recommended for identifying amyloid protein subunits to provide appropriate treatment, but it’s expensive and availability is limited [10-11]. Once the diagnosis is confirmed, cytoreduction therapy with CyBorD should be started promptly for two to four cycles followed by duratumumab maintenance for two years. Duratumumab is a monoclonal antibody that targets CD38+ plasma cells that misfold immunoglobulin light chains. It has almost doubled the complete hematological response [20]. If the treatment is partially responsive or fails, then an autologous stem cell transplant must be considered. Treatment with venetoclax is another agent used when t(11;14) is present in AL amyloidosis, but the side effects must be carefully considered [11]. Anti-fibril agents such as birtamimab and anelamimab are currently being investigated to dissolve the amyloid deposition [5].

## Conclusions

Light chain amyloidosis can present with spontaneous liver rupture as the only manifestation of the disease. Mortality is high with spontaneous hepatic rupture; therefore, stabilization and urgent CT-guided transarterial embolization are successful in most cases. Familiarity with the rare etiology of intrahepatic hemorrhage helps to decrease the high mortality rate associated with this disease. Time is crucial; therefore, workup for AL amyloidosis should not be delayed if common causes of hepatic bleeding are negative. Early diagnostic tests and treatment for AL amyloidosis can improve survival and prognosis. Recent advances in medical treatment with the addition of daratumumab to CyBorD have resulted in significant hematological response, prevention of organ deterioration, and increased patient survival. This case illustrates the importance of considering a rare disease when a patient presents with an unusual presentation.
